# Role of growth differentiation factor 11 in development, physiology and disease

**DOI:** 10.18632/oncotarget.20258

**Published:** 2017-08-14

**Authors:** Yonghui Zhang, Yong Wei, Dan Liu, Feng Liu, Xiaoshan Li, Lianhong Pan, Yi Pang, Dilong Chen

**Affiliations:** ^1^ Department of Basic Medical Science, Chongqing Three Gorges Medical College, Chongqing, China; ^2^ Chongqing Engineering Research Center of Antitumor Natural Drugs, Chongqing, China; ^3^ College of Electronic and Information Engineering, Chongqing Three Gorges University, Chongqing, China

**Keywords:** GDF11, signaling, function

## Abstract

Growth differentiation factor (GDF11) is a member of TGF-β/BMP superfamily that activates Smad and non-Smad signaling pathways and regulates expression of its target nuclear genes. Since its discovery in 1999, studies have shown the involvement of GDF11 in normal physiological processes, such as embryonic development and erythropoiesis, as well as in the pathophysiology of aging, cardiovascular disease, diabetes mellitus, and cancer. In addition, there are contradictory reports regarding the role of GDF11 in aging, cardiovascular disease, diabetes mellitus, osteogenesis, skeletal muscle development, and neurogenesis. In this review, we describe the GDF11 signaling pathway and its potential role in development, physiology and disease.

## INTRODUCTION

Growth differentiation factor (GDF11), also called as bone morphogenetic protein 11(BMP11) belongs to the transforming growth factor-β (TGF-β) superfamily. GDF11 was first reported in 1999 as a novel differentiation factor for odontoblasts [1]. Since then, many studies have investigated its distribution, structure and signaling mechanisms. Over the past 19 years, the role of GDF11 has been investigated in developmental biology and diseases such as anemia and cancer. Recent studies reported that GDF11 was a rejuvenation factor that reversed age-related heart hypertrophy and improved brain capillary and muscle function [2–4]. However, there are contradictory reports regarding the rejuvenating effects of GDF11. Restoring GDF11 in old mice had no effect on cardiac function and GDF11 had deleterious effects in aging skeletal muscle [5–6]. Additionally, there are contradictory reports regarding GDF11 function in cardiovascular disease, diabetes mellitus, osteogenesis, skeletal muscle development and neurogenesis. Therefore, in this review, we describe the GDF11 signaling pathway and its role in development, erythropoiesis, aging, cardiovascular disease, diabetes mellitus, cancer and other diseases. We also highlight the various controversies regarding the role of GDF11 in human physiology and diseases.

### Discovery, formation and distribution of GDF11

#### Discovery of GDF11

McPherron *et al.* discovered myostatin (GDF8 or MSTN), another TGF-β family member, which was closely related with GDF11 [7]. In 1999, using *MSTN* as a probe, the team cloned mouse and human *GDF11* [8]. In the same year, using rat incisor dental pulp RNA as a template and degenerate primers based on the conserved mature BMP and GDF sequences, Nakashima *et al.* showed that GDF11 was first expressed 8.5 days post-coitus, with its highest expression in tail bud, and predicted the amino acid sequences of rat and mouse GDF11 [1]. Gamer *et al.* cloned human and mouse GDF11 using a bovine BMP-related sequence to design primers and demonstrated its role in patterning in both mesodermal and neural tissues [9].

#### Structure and formation of GDF11

**Figure 1:**
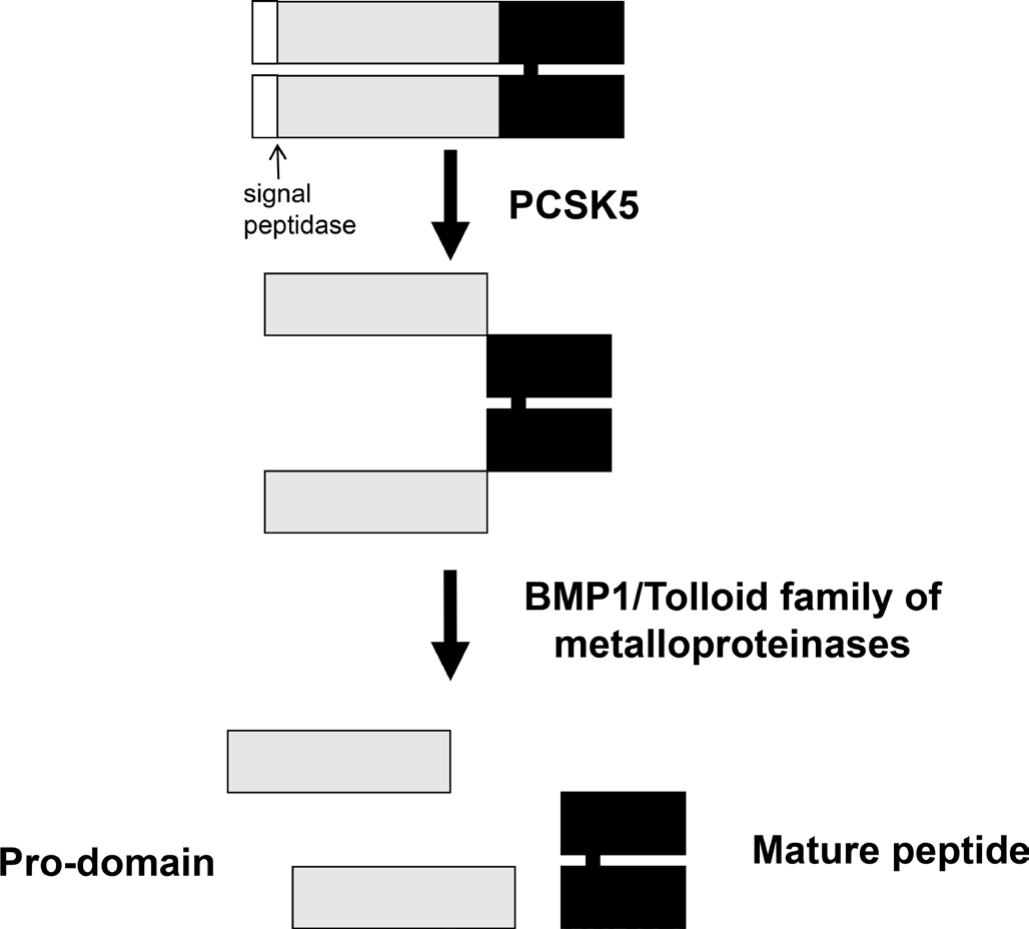
Schematic representation of GDF11 activation by sequential cleavage GDF11 is cleaved by proprotein convertase subtilisin/kexin 5/ (PCSK5) to form a non-covalent latent complex, which contains N-terminal inhibitory pro-domain and two disulfide-linked carboxyl-terminal active domain. Then, members of BMP1/Tolloid family of metalloproteinases cleave the latent complex at a single specific site to form the mature GDF11 and pro-peptide.

**Figure 2:**
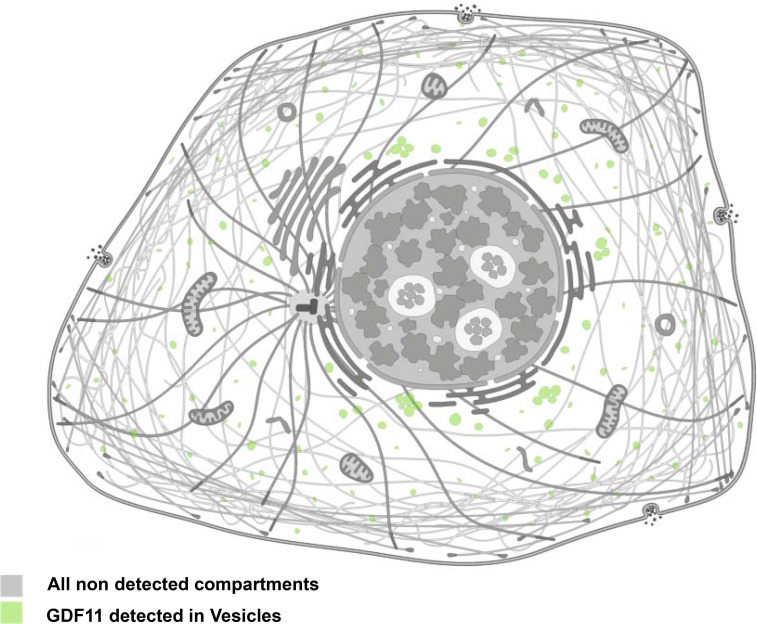
Cellular localization of GDF11 Immunofluorescence staining of GDF11 in CACO-2 and U-2 OS cell lines with anti-GDF11 antibody (Sigma-Aldrich, HPA060985; http://www.proteinatlas.org/ENSG00000135414-GDF11/cell).

The *GDF11* gene was mapped to human chromosome 12q13.2 by aligning the GDF11 sequence (GenBank AF100907) with the genomic sequence (GRCh38). It encodes a 407 amino acid protein with a signal sequence for secretion, an RXXR proteolytic processing site, and a carboxyl terminal region containing a highly conserved pattern of cysteine residues [9]. The GDF11 protein is first cleaved by pro-protein convertase subtilisin/kexin type 5 (PCSK5) to form a non-covalent latent complex, which contains N-terminal inhibitory pro-domain and two disulfide-linked carboxyl-terminal active domain [10–11]. The members of BMP1/Tolloid family of metalloproteinases then cleave the latent complex at a specific site to activate GDF11 (Figure 1). Ge *et al.* showed that the proteolytic processing of the precursor 50 kD GDF11 protein between gly119 and asp120 resulted in release of a 37 kD pro-domain and a 12.5 kD mature GDF11 [12]. The GDF11 is transported and stored in vesicles such as lipid droplets, endosomes, lysosomes and peroxisomes (Figure 2). It is possible to be translated in the rough endoplasmic reticulum, processed in the Golgi apparatus and then sorted to lysosomes, peroxisomes or secretion from the cells in transport vesicles, which needs more research to verify. Mature human, rat and mouse GDF11 have 90%, 88% and 90% amino acid sequence identity to GDF8, respectively [1, 9]. Besides, human and mouse GDF11 proteins are 99.5% identical [9].

#### Expression and distribution of GDF11

**Figure 3:**
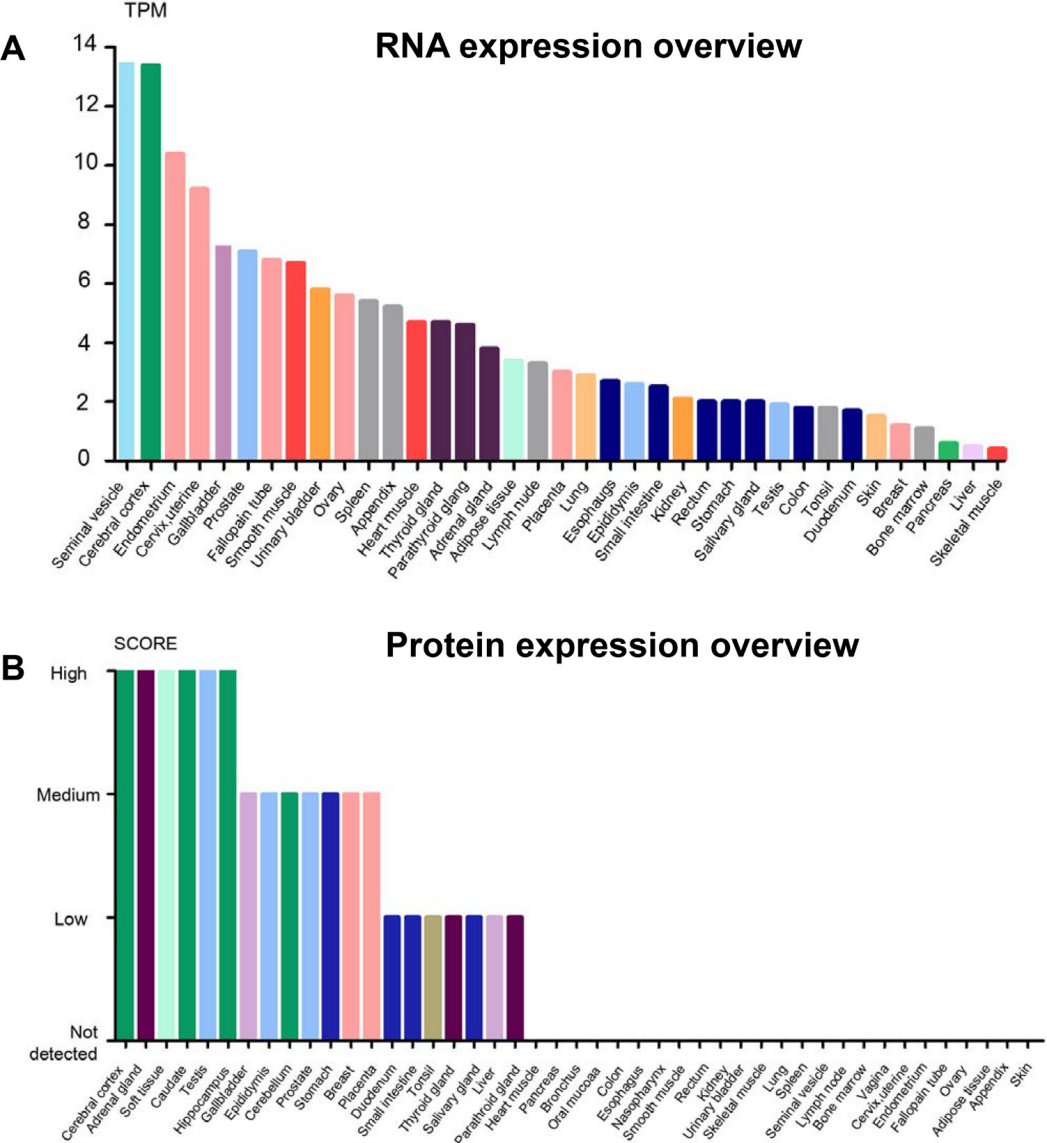
The overview of GDF11 expression in RNA and protein levels (**A**) Expression of GDF11 mRNA in human tissues from three different sources, namely, Human Protein Atlas (HPA) RNA-seq data, RNA-seq data from the Genotype-Tissue Expression (GTEx) project and CAGE data from FANTOM5 project. Color-coding is based on tissue groups that are based on tissues with common functional features. (**B**) GDF11 protein expression data is shown for 44 human tissues. The color-coding is based on tissues with common functional features. Mouse-over function shows protein score for analyzed cell types found in a selected tissue (http://www.proteinatlas.org/ENSG00000135414-GDF11/tissue).

GDF11 is expressed in embryonic tissues including tailbud, limbs and nervous system as well as adult tissues like spinal cord, olfactory system, dental pulp, skeletal muscle, spleen, pancreas, intestine, kidney, brain, heart and blood [1, 2, 9, 13, 14]. The mRNA and protein levels of GDF11 vary in different tissues. The seminal vesicle, cerebral cortex, endometrium and cervix express high amounts of GDF11 mRNA, whereas cerebral cortex, adrenal gland, soft tissue, caudate, testis and hippocampus express highest levels of GDF11 protein (Figure 3A and 3B). A recent study shows high GDF11 expression in platelets, thereby suggesting a critical platelet related function while also indicating that serum samples may not be accurate indicators of circulating GDF11 levels [15].

### GDF11 signaling pathway

**Figure 4:**
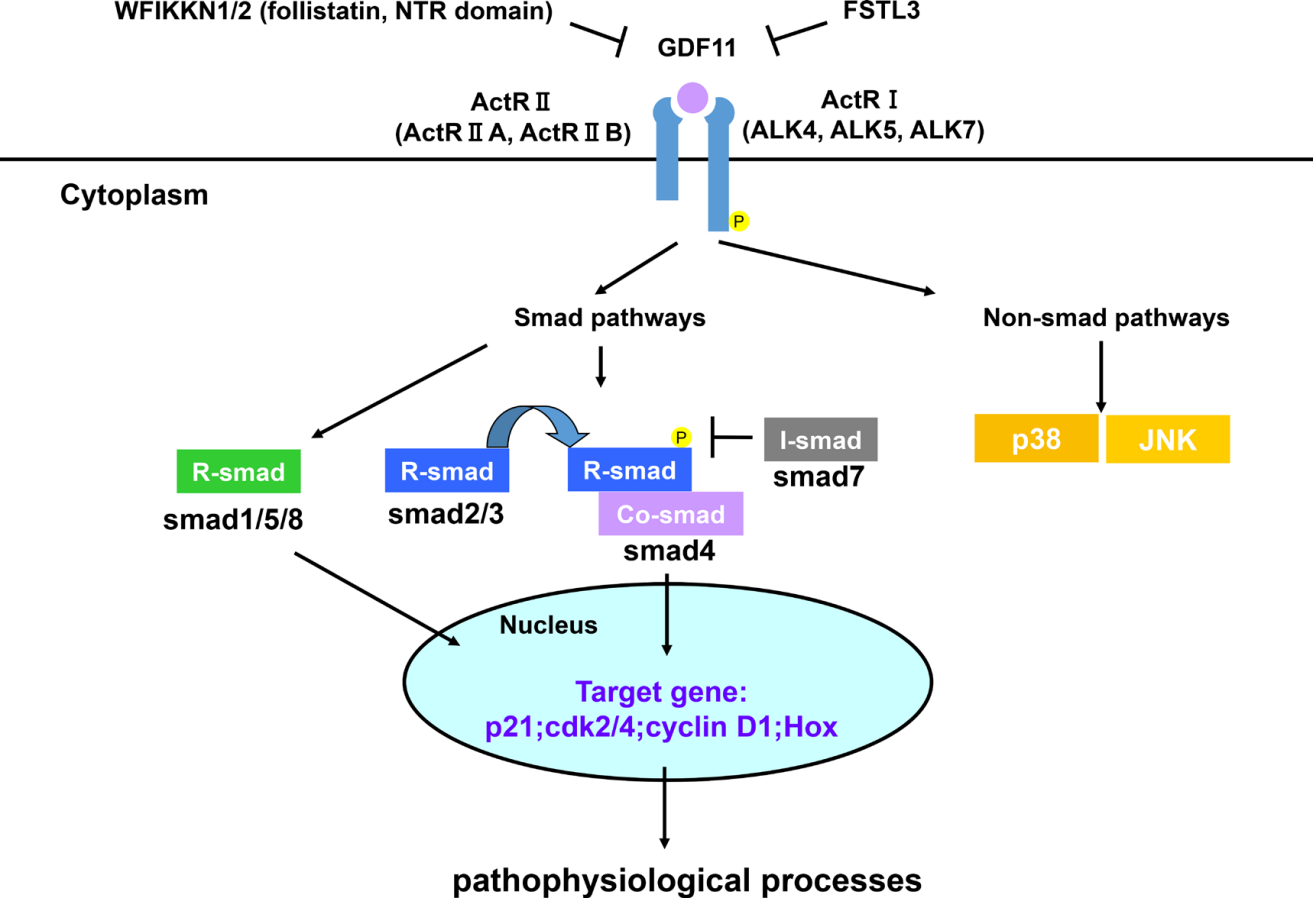
GDF11 signaling pathways GDF11 activates Smad and non-Smad signals to affect gene expression. The GDF11 signaling pathway is modulated by multiple proteins as negative feedback loops. WFIKKN1/2, FSTL3, and pro-peptide proteins inhibit GDF11 signal pathway at various points in the extracellular compartment. In the intracellular compartment, GDF11 signaling is modulated by the inhibitory Smad proteins (I-Smad, Smad7).

GDF11 transmits signals through type I and II serine/threonine kinase receptors, similar to other TGF-β superfamily members. GDF11 binds first to Activin receptor II (ActRII) including ActRIIA and ActRIIB, and then recruits Activin receptor I (ActRI) including ALK4, ALK5 and ALK7 [16, 17, 18]. GDF11 binding to its receptors activates Smad and non-Smad signaling pathways that represent canonical and non-canonical signaling pathways downstream of TGF-β superfamily members. GDF11 activates receptor-Smads (R-Smad) including Smad2/3 and Smad1/5/8. Then, they recruit common Smads (co-Smad, Smad4), migrate into the nucleus and transcribe the target genes [19–21]. Until now, most studies have focused on the Smad2/3 signaling in regard to GDF11 and only two studies have shown that GDF11 activates Smad1/5/8 in endothelial cells and chick embryos [22, 23]. Therefore, the relevance of Smad1/5/8 activation by GDF11 in the physiological and pathological processes is not clear. Besides the canonical signal, TGF-β superfamily members activate other non-Smad signals like MAP kinases (p38, ERK and JNK), Rho-like GTPase, and phosphatidylinositol-3-kinase/AKT [24, 25]. GDF11 activates p38 MAPK to regulate the size and function of nucleolus, affects JNK in endothelial cells, as well as has a crosstalk with AMPK, eNOS and NF-κB [23, 26, 27]. GDF11 signaling pathway is negatively regulated by multiple proteins. In the extracellular compartment, WFIKKN1/2, FSTL3 and pro-peptide proteins inhibit GDF11 signal pathway at various points. WFIKKN1/2 proteins, also known as GASP1/2, blocks GDF11 binding to type II receptor through its follistatin and NTR domains, thereby regulating muscle growth and development [28–29]. Follistatin-like 3(FSTL3), which is a secreted glycoprotein, forms inactive complexes with GDF11 and act as an endogenous inhibitor of GDF11 signal [30]. GDF11 propeptide antagonizes GDF11 activity *in vitro* by forming a latent complex with GDF11 [12]. In the intracellular compartment, GDF11 signaling is modulated by the inhibitory Smads (I-Smad, Smad7) [20]. In addition, histone deacetylases (HDACs), a key transcription regulator, also inhibit GDF11 gene expression to regulate the liver development in zebrafish and the growth of tumor cells [31, 32]. *In vivo* and vitro experiments, GDF11 antagonists are used to investigate the function of GDF11. GDF11 signaling pathways are summarized in Figure 4.

### Function of GDF11

#### GDF11 in development

The biology of development in animals not only involves embryogenesis, but includes regeneration, asexual reproduction, metamorphosis as well as growth and differentiation of stem cells in the adult organism. Studies have identified the role of GDF11 in spinal cord anterior/posterior patterning and development of bone, skeletal muscle, nervous system, digestive glands and urogenital system in experimental organisms and humans.

#### GDF11 in anterior posterior patterning of axial skeleton

The anterior-posterior (AP) also referred as rostral-caudal (head-tail) axis is the most ancient type of embryonic axes [33]. *GDF11* is expressed in the primitive streak and tail bud regions in the early developing mouse embryo and regulates anterior-posterior regionalization [8]. GDF11^+/−^ and GDF11^−/−^ mice show abnormalities in the axial skeleton although the phenotype is more severe for GDF11^−/−^ mice as they exhibit anterior directed homeotic transformations throughout the axial skeleton and posterior displacement of the hindlimbs. Oh *et al.* demonstrated that ActRIIA/ActRIIB compound mutant mice showed abnormal phenotypes similar to GDF11^−/−^ mice and that ActRIIA and ActRIIB mediated GDF11-related anterior/posterior patterning of the axial skeleton by activating Smad2 [34]. The heterozygous Alk5^+/−^ mice showed *GDF11*^−/−^-like phenotypes in vertebral, kidney and palate development [35]. Mechanistically, GDF11 first binds to ActRIIA and ActRIIB and then recruits type I receptor ALK5 to phosphorylate Smad2, thereby regulating the *Hox* gene expression in nucleus that influences anterior posterior patterning of axial skeleton [8, 34, 35].

#### GDF11 in the bone development

Osteogenesis (ossification) is the process of laying down new bone material by osteoblasts in bone remodeling. This involves intra-membranous and endochondral ossification. Although the exact mechanism of bone development is unclear, the TGF-β superfamily members play a vital role in the process [36, 37]. Myostatin is a critical regulator of bone development and its deficiency leads to increased bone formation and directly affects proliferation and differentiation of osteoprogenitor cells [38, 39]. Most studies show a negative role for GDF11 in osteogenesis. In postmenopausal Chinese women, GDF11 is an independent negative predictor of total hip and bone mineral density [40]. GDF11 inhibits osteoblastic differentiation of bone marrow mesenchymal stem cells via Smad2/3-Runx2 signaling, thereby inhibiting bone formation and accelerating age-related bone loss in mice [41]. Liu *et al*. showed that GDF11 led to bone loss and impaired bone regeneration in young adult and aged mice. GDF11 stimulated RANKL-induced osteoclastogenesis through Smad2/3 and c-Fos/Nfatc1, and inhibited osteoblast differentiation by repressing Runnx2 through Smad2/3 [42]. However, Zhang *et al.* showed that GDF11 was a protective factor for osteoblastogenesis by inhibiting the activity of peroxisome proliferation-activated receptor γ (PPAR-γ) [43]. Because of these conflicting results, more studies are necessary to define the role of GDF11 in bone loss-related diseases.

#### GDF11 in the muscle tissue development

Myogenesis is characterized by fusion of mononucleate myoblasts to form multinucleated myotubes, especially in embryonic development. GDF8, a secreted protein that is highly related to GDF11, is a known regulator to inhibit skeletal muscle development [44]. There are more and more researches on the role of GDF11 in the development of muscle tissue. It is reported that GDF11 inhibited muscle development and differentiation of myogenic cell lineages in chick embryos [13]. L6E9 myoblasts that lack endogenous myostatin, but express GDF11, activins, ActRIIs and follistatin have been used as *in vitro* models to identify regulators of muscle fiber size. Differentiation of L6E9 myoblasts showed decreased GDF11 level suggesting its role in muscle fiber formation [45]. Jeanplong *et al.* directly demonstrated GDF11 regulated growth of skeletal muscles through inhibiting myoblast differentiation. They found the mRNA expression of GDF11 was increased in gastrocnemius muscles at a period of rapid postnatal muscle growth and in gastrocnemius muscles of myostatin knockout mice. In addition, C_2_C_12_ myoblasts and myoblasts isolated from myostatin knockout mice were inhibited by recombinant GDF11 treatment [46]. Egerman *et al.* showed that GDF11 inhibited myoblast differentiation and muscle regeneration, thereby decreasing the number of myotubes [6]. GDF11 induced myotube atrophy by activating Smad2/3 and its overexpression led to skeletal and cardiac muscle atrophy [47]. The inhibitory function of GDF11 in skeletal muscle development was also demonstrated by GDF11 antagonists such as follistatin and GASP-2. Follistatin overexpression increased muscle weight and induced muscle hypertrophy in control as well as *Myostatin*-knockout mice, possibly by promoting satellite cell proliferation [48]. GASP-2, which is an inhibitor of both GDF11 and myostatin increased proliferation and differentiation of C_2_C_12_ myoblasts [49]. However, Sinha and others showed that GDF11 rejuvenated muscle fibers of injured old mice by increasing number of multinucleated myotubes, improving other muscle features, and restoring genomic integrity in aged muscle satellite cells [4]. Overall, most researches demonstrated GDF11 can inhibit skeletal muscle development similar to myostatin and is antagonized by factors such as follistatin and GASP-2.

#### GDF11 in the nervous system development

GDF11 is also involved in olfactory neurogenesis and optic nerve development. GDF11 and its receptors are expressed by neurons in the olfactory epithelium (OE) and their progenitors. GDF11 plays a vital role in the negative autoregulation of neurogenesis in the OE by inducing p27^Kip1^ and the reversible cell cycle arrest in progenitors [50]. OE neurogenesis is stimulated in GDF11-null mice, whereas mice with mutant GDF11 pro-domain or follistatin knockout mice dramatically decrease neurogenesis [12, 51]. The anti-neurogenic activity of GDF11 is antagonized by Foxg1, a winged-helix transcription factor, which promotes olfactory neurogenesis [52]. GDF11 and its receptor ActRIIB are expressed in the Xenopus retina and the developing mouse ganglion cell layer [1, 53]. Apart from its negative role in OE development, GDF11 also negatively regulates neuronal numbers in the retina by governing the temporal windows during which multipotent progenitors retain competence to produce distinct neural progeny independent of p27^Kip1^ levels and the effects of proliferation [54]. Santos *et al*. reported that absence of *GDF11* increased the number of retinal ganglion cells (RGCs) and reduced GDF11 levels restored retinal development in *visual system homeobox 2 (Vsx2)* deficient mice [55]. Because of its function and expression pattern in neurogenesis of optic nerve, GDF11 is a promising candidate gene in familial cavitary optic disk anomalies [56]. In addition to olfactory neurogenesis and optic nerve development, GDF11 is involved in spinal cord neurogenesis. In the developing spinal cord, GDF11 is secreted by newly born neurons and promotes cell cycle exit, decreases proliferation, changes differentiation potential and facilitates the temporal progression of neurogenesis [57]. On the other hand, Katisimpardi *et al*. demonstrated that GDF11 increased neurogenesis in aged mice. Old mice that were treated with daily doses of recombinant GDF11 increased Sox2^+^ neural stem cell populations in the brain indicating that GDF11 restored neurogenesis [3]. Therefore, the positive or negative role of GDF11 in neurogenesis may depend on different tissues and temporal contexts in an organism.

#### GDF11 in the digestive glands development

GDF11 is involved the development of liver and the islets. Histone deacetylases (HDACs) has a vital role in zebrafish liver development by regulating *GDF11* expression [31]. HDACs are required for liver and exocrine pancreas development in zebrafish. Overexpression of *HDAC3* increases liver size, whereas overexpression of *GDF11* generates a small liver phenotype although knockdown of *GDF11* alone showed no liver phenotype [31]. Meanwhile, the small liver defect in *GDF11* overexpressed embryos was neutralized by simultaneous *HDAC3* overexpression. GDF11 overexpression produced a small liver, possibly by suppressing hepatocyte proliferation. HDAC3 regulates zebrafish liver growth by inhibiting *GDF11* [31]. In the mouse, GDF11 is expressed in the embryonic pancreatic epithelium at embryonic day E12-E14 [58, 59]. GDF11^−/−^ embryos show malformation of the stomach, spleen and pancreas. Mice deficient for *GDF11* disrupt islet development and increase NGN3^+^ cell numbers, thereby indicating that GDF11 negatively regulates NGN3^+^ islet progenitor cell numbers. Moreover, GDF11 promotes pancreatic beta-cell differentiation by Smad2 in parallel to the Notch pathway [58]. In GDF11^−/−^ animals, Nkx6.1^+^ cells, which are required for development of beta cells, cannot express MafA, an insulin gene transcription factor that promotes beta cell maturation [60]. Dichmann *et al.* showed that pancreas size was two-fold reduced in GDF11^−/−^ mouse embryos with a hypoplasia of the exocrine compartment and increased NGN3+ endocrine precursor cells indicating the significant role of GDF11 in pancreatic development [59].

#### GDF11 in the urogenital system development

Kidney development follows stepwise progression of the pronephros, the mesonephros and the metanephros phases. The development process of ureteric bud, known as the metanephrogenic diverticulum originates from outgrowth of the end part of Wolffian duct, which invades into metanephric kidney induced by metanephric mesenchyme. During kidney development, GDF11 is essential for outgrowth and positioning of the ureteric bud, which is the inducer of metanephric mesenchyme [61]. GDF11 is expressed in the Wolffian duct and the metanephric mesenchyme. Mice carrying a targeted deletion of *GDF11* show bilateral or unilateral agenesis or hypoplasia of kidneys. In mesenchyme cells, GDF11 increases the expression of Glial cell line-derived neurotrophic factor (GDNF), which directs the outgrowth of a ureteric bud from the Wolffian duct [61, 62].

#### GDF11 in erythropoiesis

Erythropoiesis is the process of generating new erythrocytes and ineffective erythropoiesis is involved in anemia [63–65]. Previous studies have demonstrated that GDF11 has a critical role in normal erythropoiesis as well as the pathology of β-thalassemia, myelodysplastic syndrome (MDS), and erythropoietin-resistant anemia in hemodialysis (HD) patients [20, 66–68]. During the normal process of erythropoiesis, GDF11 is mainly expressed in immature erythroid progenitors and is necessary for their survival and to inhibit terminal differentiation [69]. In anemia, GDF11 promotes proliferation of erythroid precursors and inhibits erythroid maturation [70, 71].

Ineffective erythropoiesis and iron-restricted anemia can be treated by GDF11 traps sotatercept ACE-011 and ACE-536 [70, 71]. ACE-011 and its mouse version RAP-011, which is a ligand trap made up of the extracellular domain of ActRIIA linked to the human IgG1 Fc domain improves hematological parameters by decreasing ineffective erythropoiesis, iron overload and red blood cell-associated hemoglobin precipitates in thalassemic mice. GDF11 is overexpressed in β-thalassemia and inhibits terminal erythropoiesis [70]. ACE-536 and its mouse version RAP-536 are ligand-trapping fusion proteins containing the extracellular domain of human activin receptor type IIB modified to reduce activin binding [71]. ACE-536 increased the number of red blood cells, hemoglobin concentration and hematocrit values by promoting maturation of late-stage erythroblasts. GDF11 inhibits late-stage erythroid differentiation by activating Smad2/3. In myelodysplastic syndrome (MDS) patients, high GDF11 levels in high-risk MDS patients was accompanied by low RBC numbers, hemoglobin concentration and hematocrit values implying that GDF11 negatively correlated with late erythropoiesis [72]. In MDS erythropoiesis, GDF11 is probably involved in increased iron overload (IO) that contributes to the pathological mechanism [73]. Therefore, GDF11 and GDF11 traps are novel therapeutics in the field of aberrant iron metabolism and erythropoietic disorders.

#### GDF11 in aging and cardiovascular disease

Loffredo *et al.* reported that GDF11 reversed age-related cardiac hypertrophy [2]. Since then, GDF11 became a prominent cytokine in rejuvenation biology. However, a series of studies reported contradictory findings shown in Table 1. This has led to a debate on the role of GDF11 in aging and cardiovascular diseases.

**Table 1:** Two different viewpoints of GDF11 in aging-related cardiovascular diseases and muscle dysfunction

Viewpoints	Findings	Ref.
The positive function of GDF11 in aging-related cardiovascular diseases and muscle dysfunction	GDF11 levels decline with age.	[19] [43] [77–81]
	GDF11 reverses age-related cardiac hypertropy.	[2] [19] [77–81]
	GDF11 improves vascular and neurogenic function in aging mouse brain.	[3] [19] [78–81]
	GDF11 restores age-related skeletal muscle dysfunction in aging mouse.	[4] [19] [78–81]
	GDF11 decreases the risk of cardiovascular events and death in patients with stable ischaemic heart disease, protects against endothelial injury and reduces atherosclerotic lesion formation in apolipoprotein E-Null mice.	[27] [94]
	GDF11 serves as a novel predictor of mammalian life span.	[82]
The negative or no function of GDF11 in aging-related cardiovascular diseases and muscle dysfunction	GDF11 serum levels increase, unchange or can not be detected with age and disease.	[5, 6] [40] [84, 85]
	GDF11 does not rescue aging-raleted pathological hypertrophy.	[5] [85]
	GDF11 has a harmful effect or no significant effect on muscle function	[6] [85–87]
	Elevated GDF11 is a risk factor for age-related frailty and disease in humans.	[84] [88]
	GDF11 administration does not extend lifespan in a mouse model of premature aging.	[89]

The process of aging involves debilitating loss of tissue and cellular functions that result in degenerative pathologies. The most common features of aging include cardiac hypertrophy, decline of vascular and neurogenic function, muscle dysfunction, osteoporosis and enhanced risk of cardiovascular pathology and death. Studies have demonstrated that the mTOR inhibitor rapamycin and caloric restriction can delay aging [74, 75, 76]. Several studies showed that GDF11 reversed aging and aging-related dysfunction in muscle, nerve, cardiovascular system. While serum GDF11 levels decreased upon aging, supplementation of GDF11 rejuvenated the old mice, thereby suggesting that GDF11 was a novel predictor of mammalian life span [2, 3, 19, 43, 77–82]. GDF11 also improved tubular regeneration after acute kidney injury in elderly mice [83]. On the contrary, many studies showed that GDF11 had no significant effect on the process of aging and could not rescue aging-related pathological cardiac hypertrophy; in fact GDF11 inhibited skeletal muscle regeneration, led to bone loss in both young and aged mice and its serum levels increased or remained unchanged with age [5, 6, 40, 42, 84, 85]. Treatment with rGDF11 did not improve the dystrophic muscle pathology of mdx mutant mice, which are a model for the hereditary muscular dystrophy and it is lack of evidence for GDF11 as a rejuvenator of aged skeletal muscle satellite cells [86, 87]. Schafer *et al* reported elevated circulating GDF11 levels increased age-related frailty and disease in humans [84, 88]. Moreover, GDF11 administration did not extend lifespan of prematurely aged mice [89].

Similarly, GDF11 has a dual role in diabetes and cardiovascular diseases. Adela *et al.* demonstrated decreased plasma GDF11 levels in Type 2 diabetes mellitus (T2DM), T2DM with hypertension & coronary artery disease and T2DM with coronary artery disease in Indian patients [90]. However, Fadini *et al.* showed higher plasma GDF11 concentration in T2DM and T2DM with cardiovascular disease (CVD) patients [91]. Elevated circulating GDF11 levels increased prevalance of diabetes, prior cardiac abnormalities, frailty, risk of post-operative complications and re-hospitalization. [84, 88]. Mei *et al.* and Li *et al.* concluded that adenovirus vector transfected GDF11 and recombinant GDF11 reduced atherosclerosis in apoliprotein E^−^/^−^ mice and attenuated development of T2DM by improving islet β cell function and survival [27, 92]. However, *in vitro* experiment, Jing *et al.* showed that supplementation of GDF11 did not ameliorate the palmitate-induced insulin resistance in C2C12 myotubes [93]. In the field of cardiovascular diseases, some studies suggest that GDF11 decreases the risk of cardiovascular events and death in patients with stable ischaemic heart disease and improves aging-related cardiac hypertrophy [2, 94]. Ultrasound-targeted microbubble destruction-mediated delivery of the GDF11 plasmid to the aged heart enhanced myocardial regeneration after ischemia-reperfusion injury [95]. However, other studies concluded that GDF11 was associated with comorbidity, frailty, and post-operative outcomes in cardiovascular disease and that GDF11 supplementation did not rescue aging-related pathological hypertrophy [5, 84, 85]. In addition, the effects of GDF11 on endothelial cells are controversial as shown in Table 2. Katsimpardi *et al.* showed that GDF11 increased the proliferation of primary brain capillary endothelial cells by 22.9% for 6 day treatment in 5% FBS culture media containing VEGF and EGF [3]. However, Finkenzeller *et al.* demonstrated that GDF11 did not affect cell adherence, proliferation, and apoptosis of endothelial progenitor cells isolated from human peripheral blood in 10% FBS culture media, but increased cell migration in culture media without FBS [96]. Zhang *et al.* also showed that GDF11 did not affect cell viability, cell migration and cell proliferation of human umbilical endothelial cell and rat aortic endothelial cell in 10% FBS culture media [23]. Mei et al. concluded cell migration can not be affected by GDF11 in mice aortic endothelial cells. GDF11 did not increase cell proliferation until 5 day treatment [27].

**Table 2:** The effect of GDF11 on cell viability, cell proliferation and cell migration in different endothelial cells

Cell type	culture condition	GDF11 (concentration/time)	cell viability	cell proliferation	cell migration	Ref.
brain capillary endothelial cells	5% FBS (VEGF;EGF)	40 ng/mL 6 days		+		[3]
endothelial progenitor cells	10% FBS	40 ng/mL 3 days		none		[96]
endothelial progenitor cells	10% FBS	40 ng/mL 1 h				[96]
endothelial progenitor cells	Without FBS	40 ng/mL 1 day			+	[96]
human umbilical endothelial cells	10% FBS	50 ng/mL	none	none	none	[23]
rat aortic endothelial cells	10% FBS	50 ng/mL	none	none	none	[23]
human umbilical endothelial cells	Without FBS	50 ng/mL	+			[23]
mice aortic endothelial cells	unknown	50 ng/mL 1 or 3 days		none		[27]
mice aortic endothelial cells	unknown	50 ng/mL 5 days		+		[27]
mice aortic endothelial cells	unknown	unknown			none	[27]

+:promotion; none:no significant effect.

Therefore, there are contradictory views on the role of GDF11 in the field of aging, cardiovascular diseases, diabetes, muscle dysfunction and endothelial cell function. These may be caused by many variables in different studies such as the source of the mice, sex, recombinant GDF11 active domain, different GDF11 detection methods, the action time and concentration of GDF11, types of muscle injury, genetic background and others.

#### GDF11 in cancer and other diseases

**Figure 5:**
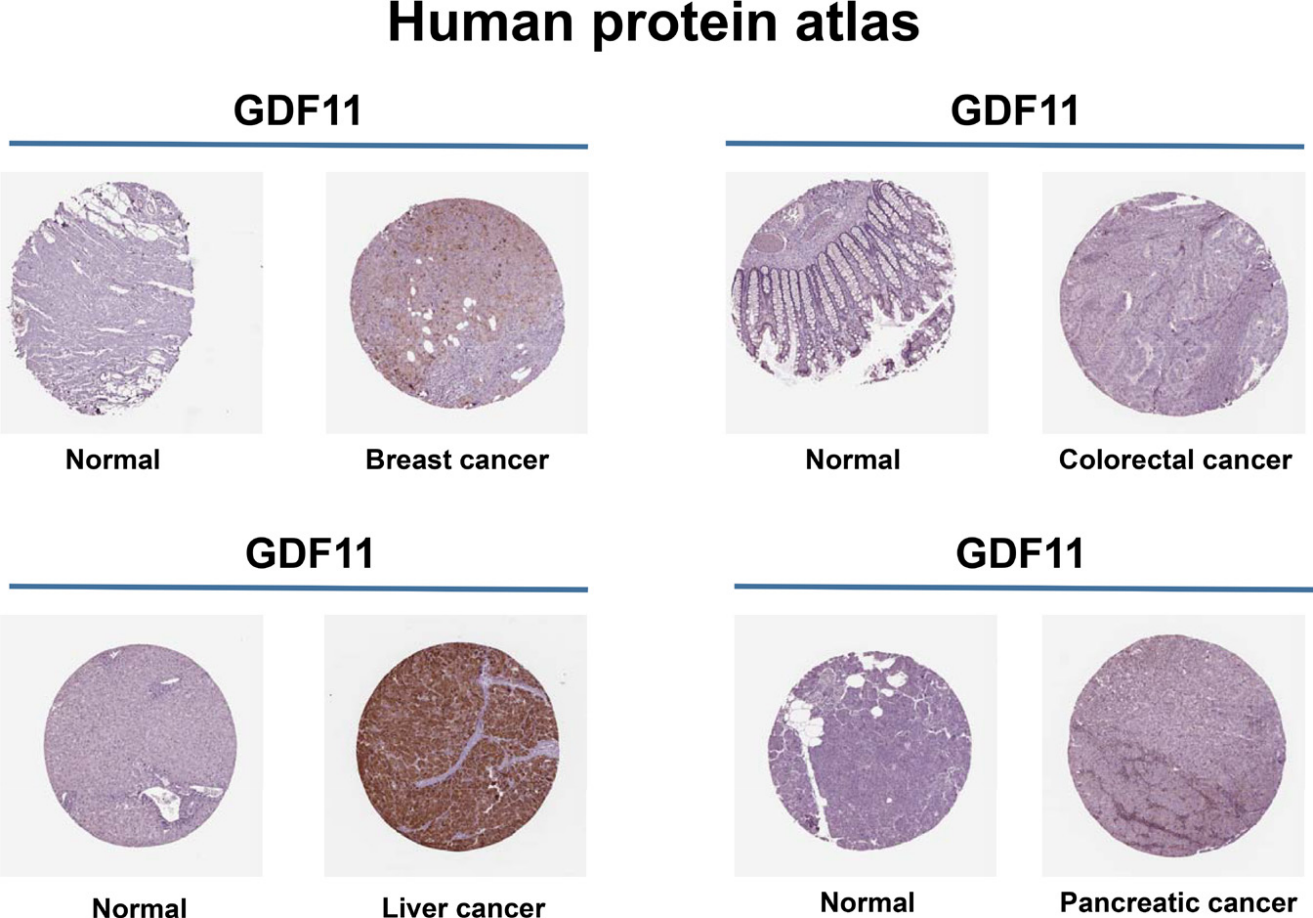
Expression of GDF11 in human cancers GDF11 expression increases in breast, colorectal, liver and pancreatic cancers. Representative images show GDF11 immunohistochemical staining of histological sections from normal and cancer tissues (Human Protein Atlas). Brown staining indicates GDF11 staining, whereas hematoxylin counterstain enables visualization of microscopical features (http://www.proteinatlas.org/ENSG00000135414-GDF11/cancer).

TGF-β family members and their receptors play a major regulatory role in cancer. In normal cells and early carcinomas, TGF-β signaling pathways exert tumor suppressor effects and in advanced tumors TGF-β signaling promotes cancer metastasis [97–99]. According the Human Protein Atlas database, GDF11 is involved in the breast, colorectal, liver and pancreatic cancers (Figure 5). Colorectal cancer patients with high GDF11 expressing tumors show a high frequency of lymph node metastasis and poor survival [100]. However, histone deacetylase (HDAC) inhibitor trichostatin A suppressed tumor growth by activating GDF11 *in vitro* [32]. The contradictory results may be caused by the dual role of TGF-β in the different stages of cancer. GDF11 is also reported to participate in breast cancer and leiomyoma uteri [101, 102]. However, GDF11 has not been investigated in other cancers and further indepth studies are needed to clarify the mechanism and function of GDF11 in cancers. Besides, GDF11 has a role in dental restoration, Epstein-Barr virus infection, primary dysmenorrhea and endometrium decidualization [103–107].

## CONCLUSIONS

In this review, we described the GDF11 signaling pathway and the role of GDF11 in physiological and pathological processes in the areas of organ development, erythropoiesis, aging, cardiovascular disease, diabetes mellitus, cancer and other diseases.GDF11 is involved in anterior-posterior pattering of axis skeleton, digestive gland development and urogenital system development. Also, most studies show that GDF11 negatively regulates osteogenesis, skeletal muscle development, olfactory and optic neurogenesis and erythroid maturation (late erythropoeisis). However, the role of GDF11 in aging and cardiovascular diseases is controversial and not comprehensively studied. Moreover, its function in most cancers and hematological disorders has also not been studied in detail. Nevertheless, studies so far demonstrate that GDF11 signaling has important implications in human development and disease with potential clinical applications in the future.
